# Standardized ileal amino acid digestibility estimates are not additive in extruded canine diets with an increasing inclusion of whole pulse flour ingredients

**DOI:** 10.1093/jas/skaf305

**Published:** 2025-08-30

**Authors:** Sydney Banton, James R Templeman, Patrícia M Oba, Kelly S Swanson, Jennifer Saunders-Blades, Anna K Shoveller

**Affiliations:** Department of Animal Biosciences, University of Guelph, Guelph, ON, CanadaN1G 2W1; Department of Animal Biosciences, University of Guelph, Guelph, ON, CanadaN1G 2W1; Department of Animal Sciences, University of Illinois, Urbana, IL, USA61801; Department of Animal Sciences, University of Illinois, Urbana, IL, USA61801; Champion Petfoods Holding Inc, Morinville, AB, CanadaT8R 1K7; Department of Animal Biosciences, University of Guelph, Guelph, ON, CanadaN1G 2W1

**Keywords:** additivity, amino acid, canine, cecectomized rooster, standardized ileal digestibility

## Abstract

The pet food industry currently formulates using crude protein (CP) and gross amino acid (AA) concentrations of individual ingredients as opposed to formulating on an AA standardized ileal digestibility (SID) basis, like in swine and poultry diets. In order to formulate on a SID basis, AA digestibility of individual ingredients must be additive when combined in a complete diet, but this assumption has not been evaluated in pet food. The objective of the present study was to determine if there is AA additivity in 4 extruded experimental diets using the AA SID estimates from the individual ingredients that were either taken from literature or determined using the cecectomized rooster. One diet contained 45% corn and corn gluten meal (Corn45), and the other 3 had increasing inclusions of whole pulse flours, up to 45% (Pulse15, Pulse30, Pulse45), all with chicken as the main protein source. To determine the AA digestibility, 28 cecectomized roosters (4 roosters per test substrate evaluated) were randomly assigned to the 4 test diets or ingredients (chicken meal, whole pinto bean flour, and whole chickpea flour). Endogenous corrections for AAs were made using 5 additional cecectomized roosters that had been fasted for 48 h. In order to determine AA SID additivity, the difference between measured and predicted SID was determined. Differences were tested for difference from zero using a one-sample 2-tailed *t*-test by the TTEST procedure of SAS (version 9.4). AA SID data in the complete diets were analyzed using the Mixed Models procedure of SAS. Of the AAs that were different among diets, the majority had greater SID estimates in Corn45 compared to the 3 pulse diets (*P* < 0.05). Only 5 AAs (tryptophan, alanine, cysteine, glutamic acid, glycine) were not different from zero when predicted SID was subtracted from measured SID in Corn45, one AA (tryptophan) in Pulse15, 2 AAs (histidine, glycine) in Pulse30, and 1 AA (glycine) in Pulse45 (*P* > 0.05). The results from this study suggest that the SID of ingredients is not additive when extruded in a complete diet intended for dogs. This highlights the need for a greater understanding of ingredient interactions in the food matrix during extrusion.

## Teaser

This study suggests that the digestibility of amino acids in individual ingredients is not predictive of the digestibility of amino acids in an extruded diet that contains a mixture of those ingredients. More information on ingredient and nutrient interactions during extrusion is necessary in order to understand how extrusion effects individual amino acid digestibility in dog diets.

## Introduction

Currently, the pet food industry formulates diets according to the amino acid (AA) composition (%) of ingredients to predict the overall AA content of the diet. However, a more informative way to formulate is to use the AA digestibility estimates, as is often done in swine and poultry nutrition. In order to do this, it is assumed that the AA digestibility estimates of the individual ingredients are additive when combined in a complete diet. This is the case in swine (Stein et al., 2005) and poultry diets (Kong and Adeola, 2013; Osho et al., 2019) since the individual ingredients are simply mixed to form a complete diet. However, in pet food, further processing, such as extrusion, is done, and heat and/or pressure can lead to nutrient interactions and affect nutrient digestibility and bioavailability (Tran et al., 2008).

The majority (65%) of the global pet food market is extruded kibble (Mordor Intelligence, 2024), despite the variety of other formats available today. Similarly, more than 50% of kibble on the market today is grain-free (Future Market Insights, 2024). Typically, grain-free diets replace cereal grain ingredients with pulse ingredients (dried legumes harvested for their seed), such as peas, chickpeas, beans, and lentils. Pulses have been used in the pet food industry as an alternative protein source for decades due to their high protein content of 17% to 30% (Boye et al., 2010). In addition, they are high in soluble fiber, giving them a lower glycemic index (Kaelle et al., 2023). This, in combination with their low-fat content, makes them potentially useful in managing weight and satiety (McCrory et al., 2010). However, pulses can contain a wide variety of anti-nutritional factors (ANFs), such as phytate, trypsin inhibitors, and tannins that can each decrease AA digestibility (Singh and Basu, 2012). In contrast, the primary ANF in corn is phytate, which primarily affects mineral absorption but can also decrease AA digestibility (Bohlke et al., 2005). Despite this, several studies reviewed by Cargo-Froom et al. (2020) report improved protein digestibility after processing methods such as extrusion, soaking, and pressure cooking, and dehulling of several pulse ingredients. In addition, pulses are also rich in oligosaccharides, stachyose, and raffinose. Studies in swine have reported decreased true and standardized indispensable AA ileal digestibility coefficients when stachyose and raffinose are at greater concentrations in soybean-based diets (Smiricky et al., 2002; Baker and Stein, 2009). However, when extruded, the oligosaccharide content of pea and lentil flour can be decreased (Berrios et al., 2010).

Standardized ileal digestibility (SID) for pulse ingredients used in pet food has been estimated previously, using the cecectomized rooster model (Reilly et al., 2020a, b). For the indispensable AAs, SID estimates of pulse ingredients ranged from ~72% to 95%, with the lowest being for methionine (Reilly et al., 2020b). In contrast, SID estimates for the indispensable AAs for corn and corn gluten meal measured in the ileal cannulated pig ranged from ~69% to 100%, with the lowest being for lysine (Almeida et al., 2011). In addition to their complementary AA profiles, pulses also contain a considerably greater amount of total dietary fiber compared to corn. The limitation with measuring the digestibility of individual ingredients is that it does not account for factors such as fiber, which, when in a complete diet, may cause nutrient interactions. For example, increasing acid and neutral detergent fiber by ~9% in swine diets can decrease true indispensable AA digestibility by up to 5% (Dilger et al., 2004).

To our knowledge, the additivity of SID values of AAs has not been determined for complete dog foods. Thus, the objective of the present study was to determine if there is AA SID additivity in 4 extruded experimental diets, one containing 45% corn and corn gluten meal (Corn45), and 3 with increasing inclusions of whole pulse flours, up to 45% (Pulse15, Pulse30, Pulse45), using the AA SID estimates from the individual ingredients that were either taken from literature or determined using the cecectomized rooster. We hypothesized that most of the AAs would not be considered additive in the complete diet due to the extrusion process and that fewer AAs would be considered additive as pulse inclusion increased due to the inherent features of pulse ingredients such as ANFs, oligosaccharides, and increased total dietary fiber.

## Materials and Methods

The protocol for the cecectomized rooster assay, including all animal housing, handling, and surgical procedures, was reviewed and approved by the Institutional Animal Care and Use Committee at the University of Illinois at Urbana-Champaign prior to experimentation (IACUC #20131).

### Diets

The diets analyzed were experimental extruded diets used in Singh et al. (2023) and Banton et al. (2024). Briefly, 4 experimental extruded diets were formulated and produced by Champion Petfoods Holding Inc. (Morinville, Alberta, CA) to meet similar macro and micronutrient targets and exceed the nutrient recommendations set out by the Association of American Feed Control Officials (AAFCO, 2021) for adult dogs at maintenance. All diets were made using a single screw extruder with similar barrel temperature ranges and dried under similar temperatures, ranging from 110 to 135 °C. The control diet contained 33% whole grain corn, 12% corn gluten meal and 0% whole pulse ingredients and the whole pulse-containing diets were formulated with 0% corn and either 5%, 10%, or 15% of each of the following: green and yellow pea flour, pinto bean flour and chickpea and lentil flour, respectively (**Table 1**). Chicken meal was used as the main animal protein source in each diet and decreased as pulse inclusion increased to achieve ~32% crude protein (CP) on an as-fed basis. All other ingredients, including vitamins and minerals, were kept the same across diets. All diets were processed and manufactured with the same ingredient batches and under similar conditions.

**Table 1.** Ingredient composition of experimental diets

	Corn45	Pulse15	Pulse30	Pulse45
**Ingredient**				
Whole grain corn	33.00	-	-	-
Corn gluten meal	12.00	-	-	-
Chicken meal	25.00	33.00	27.25	25.00
Pea starch	2.20	24.20	14.94	2.20
Whole green and yellow peas flour	-	5.00	10.00	15.00
Whole pinto beans flour	-	5.00	10.00	15.00
Whole chickpeas and lentils (50:50) flour	-	5.00	10.00	15.00
Mechanically separated chicken	10.00	10.00	10.00	10.00
Chicken fat	7.50	7.50	7.50	7.50
Ground miscanthus grass	2.00	2.00	2.00	2.00
Natural chicken flavor (dry)	1.50	1.50	1.50	1.50
Natural chicken flavor (liquid)	2.50	2.50	2.50	2.50
Salt	2.50	2.50	2.50	2.50
Potassium chloride	0.75	0.75	0.75	0.75
Kelp	0.25	0.25	0.25	0.25
Proximate analysis^1^, % as-fed basis				
Moisture	9.58	10.60	11.20	10.20
Crude protein	31.92	32.72	31.52	32.42
Crude fat	15.46	13.50	13.59	14.28
Total dietary fiber	5.50	6.65	8.21	8.31
Nitrogen-free extract (NFE, calculated)^2^	33.83	31.50	31.71	30.83
Ash	7.09	8.62	8.70	8.84
Calculated Metabolizable energy, kcal/kg^3^	3615.31	3395.32	3368.05	3427.26

All diets were formulated with equal inclusion of vitamin and mineral premixes; Vitamin B Premix Canine (0.2%), Vitamin ADE (0.2%), Choline Chloride (1.5%), Zinc (0.1%), Vitamin B5 (0.05%), Selenium (0.05%), Natural Antioxidant Liquid (0.04%), Natural Antioxidant Dry (0.02%), Copper (0.01%).

^1^Analyzed separately at the time the diets were made in May 2021.

^2^NFE = 100 - (moisture + protein + fat + fiber + ash).

^3^Metabolizable energy = ((8.5 kcal metabolizable energy (ME) x g crude fat) + (3.5 kcal ME × g crude protein) + (3.5 kcal ME × g nitrogen-free extract) × 10).

### Substrates

All 4 complete diets (Corn45, Pulse15, Pulse30, and Pulse45) were tested using the cecectomized rooster assay, as well as the chicken meal, whole pinto bean flour, and whole chickpea flour. The AA concentrations and SID values for the remaining protein-containing ingredients (whole grain corn, corn gluten meal, whole pea flour, whole lentil flour and fresh chicken) were found in the literature from either a cecectomized rooster or ileal cannulated swine model, both of which are comparable to the ileal cannulated dog (Harrison et al., 1991; Johnson et al., 1998).

### Cecectomized rooster assay

Two precision-fed rooster assays utilizing Single Comb White Leghorn roosters were conducted as described by Parsons (1985) to determine the AA SID (one for diets; one for ingredients). Prior to the studies, cecectomy was performed on roosters under general anesthesia according to the procedures of Parsons (1985). Roosters were housed individually in cages (27.9 cm wide × 50.8 cm long × 53.3 cm high) with raised wire floors. They were kept in an environmentally controlled room (approximately 24 °C, 17 h light:7 h dark).

To determine the AA SID, 28 cecectomized roosters were randomly assigned to test diets or ingredients (4 roosters per test substrate evaluated). After 26 h of feed withdrawal, roosters were tube-fed 25 g of the test substrates. Following crop intubation, excreta were collected for 48 h on plastic trays placed under each individual cage. Excreta samples were then lyophilized, weighed, and ground through a 0.25-mm screen prior to analysis. Endogenous corrections for AAs were made using 5 additional cecectomized roosters that had been fasted for 48 h.

### Chemical analyses

The test substrates and rooster excreta were analyzed for dry matter (105 °C) and ash according to the Association of Official Analytical Chemists (AOAC, 2006); method 934.01 and method 942.05. Nitrogen and CP were measured using a Leco Nitrogen/Protein Determinator (Model FP-2000, Leco Corporation, St. Joseph, MI) according to the AOAC (2006); method 982.30E. AAs were measured at the University of Missouri Experiment Station Chemical Laboratories (Columbia, MO) according to the AOAC (2006); method 982.30E using High Performance Liquid Chromatography.

### AA digestibility calculations

Basal endogenous AA concentrations were determined using roosters that were fasted for 48 h and then AA SID values were calculated by the method of Engster et al. (1985) using the equations below.


$$\begin{array}{lll} & AA\;SID\;of\;diet=\; & \frac{\left(AA\;consumed-AA\;excreted\;by\;fed\;birds+AA\;excreted\;by\;fasted\;birds\right)}{AA\;consumed}\;\times \;100\%, \end{array}$$

where,


$$\begin{array}{l} AA\;consumed\;\left(g\right)=diet\;intake\;\left(g\right)\times AA\;in\;diet\;\left(\%\right), \end{array}$$

$$\begin{array}{l} AA\;excreted\;by\;fed\;birds\;\left(g\right)=excreta\;output\;\left(g\right)\times \;AA\;in\;excreta\;\left(\%\right), \end{array}$$

$$\begin{array}{l} AA\;excreted\;by\;fasted\;birds\;\left(g\right)=excreta\;output\;\left(g\right)\times \;AA\;in\;excreta\;\left(\%\right). \end{array}$$

### Additivity calculations

The predicted SID ($SID_{Predicted})\;$was calculated according to the following equations:


$$\begin{array}{l} SID_{Predicted\;}=\frac{[(SID_{AA\;}x\;AA_{Ingredient})+{\left(SID_{AA}\;x\;AA_{Ingredient}\right)}_{n}}{{\left(AA_{Ingredient\;}+AA_{Ingredient}\right)}_{n}}, \end{array}$$

where,


$$\begin{array}{l} AA_{Ingredient}=\;\frac{AA\;concentration\times \;Inclusion\;of\;ingredient\;in\;the\;diet}{100}, \end{array}$$

and $SID_{AA}$ is the digestibility of the AA in the ingredient.

### Statistical analyses

AA SID data in the complete diets were analyzed using the Mixed Models procedure of SAS (version 9.4; SAS Institute, Cary, NC). Diet was considered a fixed effect, and rooster was considered a random effect. Tukey’s multiple comparison analysis was used to compare LS means and control for experiment-wise error. Predicted SID values were subtracted from measured SID values for each of the 4 diets, and differences were tested for difference from zero using one-sample 2-tailed *t*-test by TTEST procedure of SAS. Statistical significance was declared at *P* < 0.05.

## Results

### Chemical composition

The chemical composition and AA concentrations of test diets and ingredients are presented in **Table 2**.

**Table 2.** Chemical composition and amino acid concentrations of test diets and ingredients on a dry matter basis

	Diets				Ingredients							
Item, %	Corn45	Pulse15	Pulse30	Pulse45	Chicken meal	Whole pinto bean flour	Whole chickpea flour	Raw chicken (Oba et al., 2019)	Whole ground corn (Almeida et al., 2011)	Corn gluten meal (Almeida et al., 2011)	Whole, ground, yellow peas (Hugman et al., 2021a)	Whole, ground red lentil (Hugman et al., 2021b)
Dry matter	90.54	90.45	90.30	90.82	97.43	88.97	89.79	NR	84.11	91.03	89.27	89.20
Ash	14.78	17.48	15.89	17.30	21.08	4.77	3.70	5.84	NR	NR	6.31	2.72
Crude protein	34.55	36.32	37.04	36.13	70.74	22.99	20.13	41.72	7.94	69.08	21.13	27.69
**Indispensable AA**												
Arginine	1.80	2.17	2.18	2.22	4.86	1.18	1.65	2.22	0.39	2.48	1.66	1.78
Histidine	0.67	0.69	0.70	0.73	1.56	0.59	0.46	1.02	0.23	1.44	0.51	0.59
Isoleucine	1.31	1.37	1.37	1.41	2.80	0.99	0.85	1.75	0.27	2.86	0.93	1.09
Leucine	3.04	2.31	2.30	2.35	4.66	1.65	1.38	2.87	0.90	11.08	1.53	1.79
Total lysine	1.57	2.11	2.13	2.16	4.80	1.47	1.30	2.94	0.26	1.30	1.63	1.70
Methionine	0.69	0.57	0.56	0.53	1.44	0.26	0.26	0.87	0.17	1.77	0.20	0.19
Phenylalanine	1.43	1.35	1.37	1.42	2.53	1.18	1.07	1.42	0.37	4.43	1.05	1.22
Threonine	1.14	1.23	1.22	1.22	2.54	0.89	0.65	1.59	0.29	2.23	8.11	0.87
Tryptophan	0.19	0.26	0.21	0.29	0.28	0.11	0.12	0.44	0.05	0.48	0.20	0.14
Valine	1.51	1.62	1.59	1.63	3.11	1.13	0.87	1.88	0.38	3.17	1.03	1.20
**Dispensable AA**												
Alanine	2.21	1.90	1.86	1.83	4.71	0.88	0.79	2.01	0.56	5.82	0.93	1.01
Aspartic acid	2.41	2.86	2.92	2.98	5.69	2.40	2.07	3.22	0.52	4.23	2.42	2.67
Cysteine	0.40	0.40	0.38	0.39	0.58	0.24	0.27	0.36	0.18	1.25	0.34	0.26
Glutamic acid	4.83	4.33	4.35	4.40	9.38	3.09	2.94	4.57	1.34	13.23	3.53	3.77
Glycine	2.19	2.46	2.38	2.28	7.02	0.81	0.74	1.70	0.32	2.02	0.96	0.99
Proline	2.15	1.75	1.68	1.66	4.43	0.73	0.73	1.29	0.37	6.24	0.86	0.89
Serine	1.19	1.25	1.23	1.25	2.28	1.05	0.81	1.27	0.36	2.79	0.88	0.97
Tyrosine	1.20	0.97	1.07	1.08	2.40	0.68	0.49	1.24	0.25	3.59	0.64	0.72

NR, not reported.

### Cecectomized rooster assay

AA SID data for the diets are presented in **Table 3**. In Corn45, the majority of indispensable AA SID values were greater than 80%, with the exception of histidine and lysine. All indispensable AA SID values were greater than 70% for Pulse15, Pulse30 and Pulse45, with the SID values of arginine, methionine, and tryptophan being greater than 80%. Isoleucine, leucine, methionine, phenylalanine, valine, alanine, glutamic acid, and proline had greater SID in Corn45 compared to all pulse diets. Threonine and serine had greater SID in Corn45 compared to Pulse45 but were similar to Pulse15 and Pulse30. Cysteine and tyrosine had greater SID in Corn45 compared to Pulse15 and Pulse45, but were similar in Pulse30.

**Table 3.** Amino acid (AA) digestibility values (%) of test diets using the precision-fed cecectomized rooster assay

Amino acid	Corn45	Pulse15	Pulse30	Pulse45	SEM^1^	*P*-value
**Indispensable AA**						
Arginine	86.9	84.2	85.8	84.0	0.80	0.0732
Histidine	77.6	75.9	78.7	76.1	1.10	0.2708
Isoleucine	82.2^a^	76.7^b^	77.7^b^	76.5^b^	0.93	0.0029
Leucine	87.8^a^	77.7^b^	79.0^b^	76.9^b^	0.94	<0.0001
Lysine	76.1	77.9	78.1	76.9	1.06	0.5178
Methionine	86.7^a^	81.7^b^	82.6^b^	79.7^b^	0.86	0.0007
Phenylalanine	85.0^a^	76.5^b^	77.2^b^	75.5^b^	1.03	0.0001
Threonine	80.1^a^	76.5^ab^	78.9^ab^	74.4^b^	1.24	0.0288
Tryptophan	90.2	91.3	91.5	91.8	0.74	0.4408
Valine	81.4^a^	75.6^b^	76.6^b^	74.7^b^	1.11	0.0048
**Dispensable AA**						
Alanine	85.7^a^	77.1^b^	78.7^b^	76.9^b^	0.96	<0.0001
Aspartic acid	74.7	71.7	74.6	71.6	1.09	0.1013
Cysteine	70.2^a^	58.5^b^	64.3^ab^	58.6^b^	1.98	0.0035
Glutamic acid	85.9^a^	79.0^b^	80.^6b^	78.4^b^	0.87	0.0002
Glycine	76.4	69.3	71.2	70.2	2.99	0.3805
Proline	86.3^a^	76.9^b^	79.9^b^	76.1^b^	1.13	0.0001
Serine	81.5^a^	75.1^ab^	76.6^ab^	71.4^b^	1.59	0.0056
Tyrosine	84.1^a^	74.8^b^	79.1^ab^	76.5^b^	1.24	0.0011

^1^Pooled standard error of the mean.

^a,b^Means with different superscripts within a row differ by Tukey’s test (*P* < 0.05).

AA SID data for the test ingredients, as well as the ingredients found in literature, are presented in **Table 4**. For whole chickpea flour and chicken meal, the majority of indispensable AA SID values were greater than 80%, with the exception being tryptophan in whole chickpea flour. All indispensable AA SID values for whole pinto bean flour were greater than 50%, with arginine, histidine, lysine, and threonine being greater than 60%.

**Table 4.** Amino acid (AA) digestibility values (%) of test ingredients and ingredients found in literature

Amino Acid	Chicken meal	Whole pinto bean flour	Whole chickpea flour	Fresh chicken (Oba et al., 2019)	Whole ground corn (Almeida et al., 2011)	Corn gluten meal (Almeida et al., 2011)	Whole, ground, yellow peas (Hugman et al., 2021b)	Whole, ground red lentil (Hugman et al., 2021a)
**Indispensable AA**								
Arginine	90.9	93.5	68.1	88.9	100.1	93.7	90.0	86.2
Histidine	85.4	81.3	62.1	79.8	83.7	82.8	86.9	79.7
Isoleucine	90.5	84.3	56.3	90.8	80.9	86.4	83.5	77.8
Leucine	90.7	85.8	58.4	90.7	88.0	91.3	82.4	79.0
Lysine	85.2	83.7	66.3	86.6	69.2	78.7	87.5	80.6
Methionine	91.3	84.4	57.6	93.4	86.2	90.6	84.8	75.6
Phenylalanine	89.7	88.7	56.2	88.8	85.9	89.4	84.2	78.7
Threonine	89.1	85.3	62.0	84.6	74.9	83.5	80.4	79.0
Tryptophan	92.1	80.0	50.4	94.2	83.9	91.8	77.3	86.4
Valine	89.2	83.7	57.0	86.3	80.1	84.9	80.9	77.3
**Dispensable AA**								
Alanine	87.5	79.2	56.2	88.8	86.6	87.8	81.5	77.5
Aspartic acid	78.6	88.9	58.6	88.9	77.2	83.2	83.8	81.3
Cysteine	76.3	80.1	60.0	50.2	79.6	81.0	74.8	71.7
Glutamic acid	88.4	91.3	65.9	87.4	87.7	87.6	87.5	82.2
Glycine	78.0	58.9	31.6	57.6	107.4	66.5	82.8	79.2
Proline	86.1	86.9	57.3	77.6	193.3	97.2	91.9	80.6
Serine	86.8	88.5	61.0	79.7	85.1	89.7	81.0	80.2
Tyrosine	88.1	80.7	56.9	84.2	84.8	89.6	84.6	80.2

### Additivity

The differences between measured and predicted SID are presented in **Table 5**. Five AA were not different from 0 (*P* > 0.05) when the predicted SID was subtracted from the measured SID for Corn45 (tryptophan, alanine, cysteine, glutamic acid and glycine), one AA was not different from 0 for Pulse15 (tryptophan), 2 AA were not different from 0 for Pulse30 (histidine and glycine) and one AA was not different from 0 for Pulse45 (glycine). In contrast, most AA were different from 0 when the predicted SID was subtracted from the measured SID. For Corn45, arginine, histidine, isoleucine, leucine, lysine, methionine, phenylalanine, threonine, valine, aspartic acid, proline, serine, and tyrosine were different from 0 (*P* < 0.05). For Pulse15, arginine, histidine, isoleucine, leucine, lysine, methionine, phenylalanine, threonine, valine, alanine, aspartic acid, cysteine, glutamic acid, glycine, proline, serine, and tyrosine were different from 0 (*P* < 0.05). For Pulse30, arginine, isoleucine, leucine, lysine, methionine, phenylalanine, threonine, tryptophan, valine, alanine, aspartic acid, cysteine, glutamic acid, proline, serine, and tyrosine were different from 0 (*P* < 0.05). For Pulse45, arginine, histidine, isoleucine, leucine, lysine, methionine, phenylalanine, threonine, tryptophan, valine, alanine, aspartic acid, cysteine, glutamic acid, proline, serine, and tyrosine were different from 0 (*P* < 0.05).

**Table 5.** Difference between measured and predicted digestibility for amino acids (AA) in each of the 4 test diets (Corn45, Pulse15, Pulse30, Pulse45)

	Corn45		Pulse15		Pulse30		Pulse45	
	Difference	SEM	Difference	SEM	Difference	SEM	Difference	SEM
**Indispensable AA**								
Arginine	−4.8	0.7	−5.8	0.5	−3.4	0.8	−4.6	1.1
Histidine	−6.2	1.0	−7.5	0.6	−3.5^*^	1.7	−5.3	0.8
Isoleucine	−6.6	0.8	−11.9	0.2	−8.8	1.2	−8.3	1.2
Leucine	−2.9	0.7	−11.1	0.3	−7.8	1.1	−8.3	1.3
Lysine	−8.0	1.0	−6.8	0.9	−5.9	1.4	−6.5	0.9
Methionine	−4.3	0.8	−8.9	0.2	−6.7	1.0	−8.5	1.1
Phenylalanine	−4.2	0.7	−10.8	0.2	−7.9	1.2	−8.0	1.5
Threonine	−5.9	1.4	−8.7	0.4	−4.3	1.2	−7.7	1.6
Tryptophan	−1.7^*^	0.7	1.3^*^	0.5	4.1	1.1	6.5	0.6
Valine	−5.5	1.1	−11.2	0.4	−8.2	1.3	−8.4	1.4
**Dispensable AA**								
Alanine	−2.0^*^	0.7	−9.5	0.2	−6.6	1.2	−7.3	1.3
Aspartic acid	−6.1	1.5	−7.8	1.1	−4.5	0.8	−7.1	1.2
Cysteine	−6.0^*^	2.3	−13.5	1.5	−7.0	2.1	−12.4	1.9
Glutamic acid	−2.1^*^	0.7	−8.3	0.4	−5.7	1.0	−7.1	1.2
Glycine	−0.2^*^	4.1	−6.6	1.2	−3.6^*^	2.1	−3.7^*^	3.6
Proline	−9.4	1.0	−8.0	1.0	−4.3	1.3	−7.5	1.2
Serine	−5.2	1.5	−9.1	1.1	−6.0	1.5	−10	2.1
Tyrosine	−3.9	1.0	−11.4	0.8	−5.6	1.5	−7.0	1.5

^*^ *P* > 0.05 indicating value is not different from 0.

## Discussion

To the author's knowledge, this is the first study to assess if AAs are additive when formulating based on SID and when ingredients are mixed and undergo extrusion. In contrast to other species fed mixed diets, the majority of AAs in the extruded diets used in this study were not additive, providing valuable insight into the potential to use AA digestibility estimates in the formulation of dog and cat diets.

The majority of AAs were more digestible in the Corn45 diet compared to the pulse-inclusive diets. Interestingly, of the indispensable AAs, arginine, lysine, and tryptophan were numerically greater in the pulse-containing diets, yet there was no difference in their digestibility among all diets. In contrast, leucine and methionine were numerically greater in the Corn45 diet, and their digestibility was greater in the Corn45 diet compared to the 3 pulse diets. This highlights that greater dietary concentrations do not necessarily mean greater digestibility. There are limited papers that directly compare the AA digestibility of pulse ingredients versus grain ingredients, and none that do so for ingredients intended to be fed to dogs. However, one paper reports the apparent ileal digestibility (AID) coefficients in broiler chickens fed various grain and pulse ingredients, but it is unclear if the ingredients were processed in any way (Ravindran et al., 2005). Although not statistically compared, the AID values for corn were generally greater than the AID values for chickpeas, faba beans, and field peas (Ravindran et al., 2005). In addition, increasing total dietary fiber in a complete diet can also decrease AID and SID of most AAs (Dilger et al., 2004; Chen et al., 2015; Fouhse et al., 2017), likely due to an increase in endogenous nutrient losses. A limitation of the present study and those listed above is that specific AA losses were not measured, which are known to be induced by fiber (Ravindran, 2016). Dietary fiber can increase mucin production (Satchithanandam et al., 1996), which is one of the contributors to endogenous AA losses. For example, the greatest AA concentration in mucin is threonine. In pigs fed a diet that replaced a portion of the cornstarch with either barley or wheat bran (moderate and high in hemicellulose, respectively), threonine had the lowest AID but no difference in SID and had the greatest total ileal flow of threonine compared to the control casein diet (Myrie et al., 2008). This suggests an increase in endogenous losses of threonine, likely from increased mucin production in pigs fed a diet containing barley or wheat bran (Myrie et al., 2008). Dietary fiber has also been shown to increase fecal excretion of primary bile acids in dogs fed a grain-free diet with a 26% inclusion of peas compared to dogs fed a grain-based diet (Pezzali et al., 2020). However, very limited data exists on the specific AA losses induced by feed ingredients due to the complexity of the methodology required to measure specific losses (Ravindran, 2016). The total dietary fiber increased as pulse inclusion increased in the present diets, which likely played a role in the lower SID values observed in the pulse diets compared to Corn45. Another explanation may be the use of corn gluten meal in the Corn45 diet. This diet used both whole ground corn and corn gluten meal; however, corn gluten meal is the protein-rich by-product after the starch and germ have been removed. This processing makes it a highly digestible source of AAs (Almeida et al., 2011). In contrast, the pulse diets used whole flours where the starch and other plant cell components would still be present and contribute to ingredient interactions that may decrease digestibility.

All AAs across all diets, except for tryptophan in the pulse-containing diets, had negative values when the predicted SID was subtracted from the measured SID, suggesting that the predicted SID was greater than the measured SID. This negative value means that if the SID values of the individual ingredients were used to estimate the SID of the complete diet, most AAs would be overpredicted, suggesting that the AAs in the complete diet are more digestible than they actually are. Tryptophan, having a positive difference in the pulse diets, suggests that the measured tryptophan was greater than the predicted. Tryptophan is measured using a base hydrolysis technique that is different from the acid hydrolysis that is used to determine other AAs (Rutherford and Moughan, 2012). This often leads to a lower recovery of tryptophan. It is possible that the tryptophan content of the individual ingredients is underestimated, leading to an underestimate of the predicted SID. However, this corresponded to tryptophan being considered additive in 2 of the 4 diets due to the difference between measured and predicted SID not being different from 0. More AAs were additive in the Corn45 diet compared to the pulse-containing diets, suggesting that the addition of pulses decreases AA SID additivity, as hypothesized.

Glycine was considered additive in 3 out of 4 diets. Early work investigating the effect of heat processing on AA damage reported little to no destruction of glycine after autoclaving soybean products and measured via acid hydrolysis (Evans et al., 1951). In comparison, in a similar study, 30% of lysine and 40% of cysteine were destroyed after autoclaving (Evans and McGinnis, 1948), suggesting that glycine is more stable during heat processing and easily recovered via acid hydrolysis, which is still used today to measure glycine. This is likely why the measured and predicted glycine were similar in 3 of the 4 diets. Although the AOAC methods for AA analysis are what are currently used to measure AAs in pet food, these analytical techniques can be highly variable. One study measuring the AA in poultry meal reported the repeatability relative standard deviation as 0.8% to 12.7% and reproducibility relative standard deviation as 3.7% to 24.1% across all AAs (Llames and Fontaine 1994). This variability could have also contributed to the lack of AA SID additivity in most of the AA reported here and emphasizes the need for innovation in AA analysis for more accurate results.

Several studies suggest that the AA SID of individual ingredients are additive when combined in a complete diet (Stein et al., 2005; Kong and Adeola, 2013; Osho et al., 2019). However, these have been done in swine and poultry, where the complete diets are only mixed and not processed further. One study that examined AA AID additivity in pelleted fish diets with steam pretreatment reported Index of Similarity values from 95% to 105% for the indispensable AA, thus considering them all additive (Lupatsch et al., 1997). Although the temperature during pelleting is lower than during extrusion, it can still cause protein denaturation, formation of Maillard products, and cross-linked AA, similar to extrusion (Svihus and Zimonja, 2011). However, these outcomes would affect bioavailability measures, not digestibility. Pelleting can also increase the SID of AAs in a complete diet fed to ileal cannulated swine (Lahaye et al., 2008). However, other than wheat flour and soybean meal, the individual ingredients used by Lupatsch et al. (1997) were all meat meals that had been cooked and ground prior to pelleting; therefore, there were likely fewer nutrient interactions, such as those with fiber. As noted earlier, the individual ingredients that were analyzed were uncooked and ground; however, the final diet was extruded, as is often the case for dog food. Therefore, the AA content and SID from the uncooked, ground ingredients were used to calculate the predicted SID. Extrusion can increase the SID of peas and lentils (Hugman et al., 2021a, b). However, if AA SID values for extruded ingredients were used to calculate predicted SID, then it is assumed that the predicted SID would be even higher than the results we present here. Therefore, it is likely that ingredient interactions are occurring during processing of the complete diet. It is well known that extrusion can lead to gelatinization of starch, denaturation of proteins, decreases in ANFs, and the formation of Maillard products and cross-linked AA (Reviewed in Tran et al., 2008). Some of these ingredient interactions during extrusion could play a role in the additivity of the AAs in the present diets.

In terms of starch, it is important to note that Pulse15 and Pulse30 had considerably more pea starch in order to match energy and protein targets among diets. Pulse starches have higher amylose: amylopectin compared to cereal starches (Ren et al., 2021), which may increase resistant starch, which can evade enzymatic digestion (Du et al., 2014). In fact, the AID values in ileal cannulated pigs fed sticky rice, maize, or resistant starch diets were significantly lower for all indispensable AAs in pigs fed the resistant starch diets (Yin et al., 2010). This may help explain why the pulse diets in the present study had fewer AAs that were considered additive compared to the Corn45 diet. More specifically, in terms of the difference between measured and predicted SID, the Pulse15 diet had the most AAs with a difference greater than 10 (isoleucine, leucine, phenylalanine, valine, cysteine, tyrosine). In fact, no other diet had a difference greater than 10 for any AA except for Pulse45 for cysteine. The Pulse15 diet had the greatest amount of pea starch across all diets, which could have led to the greater differences in more AAs. A limitation of the present study is that the resistant starch was not analyzed in the diets; however, starch cook was, and the percent of total starch as cooked starch was greatest for Pulse15 (95%), followed by Pulse45 (91%), Pulse 30 (89%), and Corn45 (75%; data not shown). This aligns with previous work that reported greater starch cooking in commercial grain-free extruded diets compared to commercial grain-inclusive extruded diets, yet the grain-free diets still had numerically greater resistant starch (Corsato Alvarenga and Aldrich, 2020). Although Pulse15 had greater starch cook, it may have still had greater resistant starch in comparison to Corn45. Although cooking can decrease resistant starch, resistant starch was 16% to 30% in isolated pea starches from 9 Canadian pea varieties and lower in cooked corn starch (10%) than in 5/9 of the pea varieties (Lu et al., 2022).

Denaturation of proteins during extrusion can increase the digestibility of AAs, and this is supported in dog diets that were formulated with yellow peas, green lentils, or garbanzo beans as the main protein sources, where SID values were numerically greater after extrusion compared to the pre-extruded mixture of ingredients (Hsu et al., 2024). Extrusion also significantly reduced, to the point of negligible concentrations, the ANFs present in the pulse diets (Hsu et al., 2024). Uncooked pulse ingredients contain several ANFs that can be toxic, unpalatable, or decrease AA digestibility; however, extrusion can significantly reduce these. Extrusion decreases trypsin inhibitors, tannins, and phytate activity in many pulses, like peas, chickpeas, faba, and kidney beans (El-Alonso et al., 2000a, 2000b; Abd Hady et al., 2003). Although the ANFs were not measured in the current diets, extruded pulse-containing diets fed to dogs contain negligible amounts of trypsin inhibitor activity (Reilly et al., 2021). Therefore, it is unlikely that ANFs would be a significant contributor to the lack of additivity of AAs in the present study. However, ANFs could have been a significant contributor to underestimating the predicted SID of the pre-extruded pulse ingredients. But again, theoretically, this would not have improved our additivity scores as the predicted was already greater than the measured SID in the results presented here.

Maillard reactions occur between lysine and reducing sugars, among others, where the reducing sugar binds to the epsilon amino group of lysine, making lysine metabolically unavailable to the animal, and this is accelerated during heat processing and cooking (Hurrell and Carpenter, 1974). As this product can still be absorbed by the animal but not used, this does not affect lysine digestibility, which is what was measured in the present study, but instead affects lysine bioavailability, an outcome not investigated herein. Extrusion can also lead to cross-linkages of AAs. Of particular interest to the present study, alanine can be converted to dehydroalanine, which can then react with cysteine to form lanthionine (Hurrell et al., 1976). In the extruded pulse diets used in Hsu et al. (2024), while the majority of AA concentrations numerically increased after extrusion compared to the uncooked ingredients, the cysteine concentrations numerically decreased. This may help to explain the greater difference in measured versus predicted SID values for cysteine in the Pulse15 and Pulse45 diets. However, it is unclear why this difference was not as drastic in the Corn45 and Pulse30 diets, as all diets were extruded similarly.

The measured cysteine SID was much lower in the Pulse15 and Pulse45 diets compared to the Corn45 diet at only ~58%. Of interest to some of the literature surrounding taurine deficiency and grain-free diets (Kaplan et al., 2018; Freid et al., 2021), none of the dogs fed the present diets in Singh et al. (2023) had low or deficient fasted plasma or whole blood taurine concentrations, despite the low cysteine SID. In fact, there were no treatment differences among any diet for fasted plasma methionine, cysteine, or taurine concentrations (Singh et al., 2023). Cysteine is the direct precursor to taurine and its concentration influences taurine synthesis more so than methionine (Stipanuk and Ueki, 2011). All of these diets oversupplied methionine and cysteine using ingredients and no synthetic supplemental AAs, likely allowing adequate taurine synthesis despite having a low cysteine SID. In addition, these diets oversupplied protein at ~32%; therefore, even though the measured SID was lower than the predicted for most AAs, this likely would not negatively affect the animal. Where this may become a problem is in a lower protein diet or a diet that is just meeting AA requirements, and a lower digestibility than predicted may lead to a deficiency if fed long-term. For example, Sanderson et al. (2001) fed 3 protein-restricted (~10% on a dry matter basis) diets to adult beagles for 48 mo and by 18 mo, the mean whole blood taurine concentration of each group was considered to be deficient. Although the methionine and cysteine concentrations in the diets were above AAFCO, the other AA concentrations in the diets were not disclosed, nor was digestibility measured. However, this highlights the need for careful formulation of diets, especially when using ingredients that are known to be limiting in specific AAs, such as the case with pulses and methionine, or in lower protein diets.

The results from this study highlight the need for a greater understanding of ingredient interactions in the food matrix during extrusion. Currently, we cannot conclude that AAs of pre-extruded ingredients are additive when extruded in a complete diet intended for dogs. If this were the case, however, we could use the AA digestibility of individual ingredients when formulating complete diets, as is often done in swine nutrition. This would greatly improve our ability to provide predicted digestibility of dog and cat foods and reduce the use of animals in digestibility trials. In order to do this, future research could start by investigating how ingredients perform in combination, such as a protein and fiber source, both before and after extrusion, in terms of AA digestibility. Further, it would be of interest to understand how different extrusion conditions change the AA digestibility of pairs of ingredients. This is not an easy task, especially given the diversity of ingredients used in pet food today and the almost unlimited number of ingredient combinations and extrusion parameters. However, formulating using SID estimates would allow more precise protein/AA delivery and less oversupply of protein, which could help make the pet food industry more sustainable. However, steps need to be taken to begin to understand ingredient interactions in the food matrix that can be more widely applied to a variety of diets.

## List of Abbreviations

AA: Amino acid
ANFs: anti-nutritional factors
AID: apparent ileal digestibility
AAFCO: Association of American Feed Control Officials
AOAC: Association of Official Analytical Chemists
SID: standardized ileal digestibility
